# Association between socioeconomic factors and semaglutide use for weight loss: a population-based cross-sectional study in Denmark

**DOI:** 10.1016/j.lanepe.2025.101398

**Published:** 2025-07-28

**Authors:** Rasmus Bo Hasselbalch, Mille Kyhn Andrea, Carl Villaro Nolsøe, Mathias Hindborg, Puriya Daniel Würtz Yazdanfard, Kathrine Kold Sørensen, Stig Nikolaj Fasmer Blomberg, Helle Collatz Christensen, Claus Graff, Cæcilie Stilling Denholt, Shoaib Afzal, Børge G. Nordestgaard, Kristian Kragholm, Henning Bundgaard, Kasper Karmark Iversen, Christian Torp-Pedersen, Mikkel Porsborg Andersen

**Affiliations:** a Department of Cardiology, Copenhagen University Hospital - Rigshospitalet, Copenhagen, Denmark; b Department of Cardiology, Copenhagen University Hospital - Herlev and Gentofte Hospital, Copenhagen, Denmark; c Department of Pathology, Copenhagen University Hospital - Herlev and Gentofte Hospital, Copenhagen, Denmark; d Department of Cardiology, Nordsjællands Hospital, Hillerød, Denmark; e Prehospital Center, Region Zealand, Næstved, Denmark; f Department of Health Science and Technology, Aalborg University, Aalborg, Denmark; g Department of Clinical Biochemistry and the Copenhagen General Population Study, Copenhagen University Hospital - Herlev and Gentofte Hospital, Copenhagen, Denmark; h Department of Clinical Medicine, Faculty of Health and Medical Sciences, University of Copenhagen, Copenhagen, Denmark; i Department of Cardiology, Aalborg University Hospital, Aalborg, Denmark; j Department of Clinical Medicine, Aalborg University, Aalborg, Denmark; k Department of Public Health, University of Copenhagen, Copenhagen, Denmark

**Keywords:** Obesity, Income disparity, Semaglutide

## Abstract

**Background:**

Glucagon-like peptide-1 agonists like semaglutide are effective treatments for obesity. High costs may create economic barriers. This study examines the association between income and prescriptions for semaglutide for weight loss.

**Methods:**

This nationwide register-based cross-sectional study of all adults in Denmark without diabetes, analyzed the association between household income adjusted for family size divided into quartiles and semaglutide prescription redemption for weight loss. We obtained weight and height on a representative sample of the population from The Copenhagen General Population Study which randomly selected individuals in 2014–2019. Obesity was defined as a body mass index >30 kg/m^2^.

**Findings:**

A total of 4,531,146 adult individuals were included after excluding individuals with diabetes (186,823, 3·8%), and individuals without income data (146,639, 3·0%). The proportion of individuals with a redeemed semaglutide prescription increased with income, from 1·3% (n = 13,925) in the lowest income quartile to 3·6% (n = 41,298) in the highest. Conversely, in a representative sample of 36,391 individuals, the proportion living with obesity was 26% (n = 1310) in the lowest income quartile compared to 13% (n = 1872) in the highest. During the year 2023 we found a general increase in Semaglutide use from 40,605 (0·9%) in the first quarter of 2023 to 85,250 (1·9%) in the fourth quarter, which was most pronounced in women in the highest income group with an increase from 10,818 (1·9%) to 23,069 (4·1%).

**Interpretation:**

Semaglutide use increased with income while obesity declined. This suggests that economic concerns lead to a systematic undertreatment for obesity for low-income individuals, potentially exacerbating existing health inequalities.

**Funding:**

None.

**Research in context**

**Evidence before this study**

Glucagon-like peptide-1 receptor agonists (GLP-1RA) like semaglutide are effective treatments for obesity. However, high costs may create socioeconomic barriers and increase inequality in health. This study examines the association between income and prescriptions of semaglutide for weight loss. We searched PubMed for relevant publications up to April 9, 2025 using the following search terms: (“socioeconomic factors” [Mesh] or “social class” [Mesh] or “socioeconomic” [tiab] or “social” [tiab] or “income” [tiab] or “education∗” [tiab] or “depriv∗” [tiab]) and (“Glucagon-Like Peptide 1” [Mesh] or “glp1∗” [tiab] or “glp-1∗” [tiab] or “glucagon-like-peptide-1” [tiab]) and (“Weight loss” [Mesh] or “Obesity” [Mesh]). Few studies have investigated socioeconomic differences in initiation of GLP-1RA for weight loss. A Danish study of patients with diabetes found that GLP1-RA was more common for high income individuals (11·4% vs. 9·5% for highest versus lowest income group) despite GLP1-RA being reimbursed for this indication. However, since GLP1-RA was not approved for weight loss at the time, the association was not investigated for this indication. A recent Swedish study of around 16,000 individuals with a prescription for GLP1-RA showed that off label use of GLP1-RA was more common among males with a higher income.

**Added value of this study**

This study is the first to investigate nationwide patterns of semaglutide use for weight loss among individuals without diabetes in Denmark, with a specific focus on socioeconomic disparities. In a population of over 4·5 million adults, we found a strong socioeconomic gradient in prescription rates; only 1·3% of individuals in the lowest income quartile received semaglutide, compared to 3·6% in the highest quartile. Notably, this pattern was observed despite our subsequent finding in a subpopulation that a substantially higher prevalence of people living with obesity in the lowest income group (26% vs. 13%). These associations remained robust after adjusting for age, sex, educational attainment, immigration status, and comorbidities.

**Implications of all the available evidence**

The growing availability of effective anti-obesity medications like semaglutide offers promising new avenues for addressing a major public health burden. However, our findings suggest that access to these treatments may currently be limited by socioeconomic factors, with individuals from lower-income groups, despite a higher burden of obesity, being less likely to receive them. This disparity could contribute to an increase in health inequality unless policy measures are implemented to ensure equitable access. Our study underscores the urgent need to consider affordability and equity in the broader implementation of pharmacological obesity treatments.

## Introduction

Obesity is a global public health issue with increasing prevalence despite persistent public health efforts.^1^ The proportion of adults living with obesity has risen significantly in recent decades, with approximately 18·7% of the adult Danish population currently affected.^2^ This growing epidemic is linked to severe health consequences, including elevated risks of chronic diseases such as type 2 diabetes, cardiovascular conditions, and cancer.^1^^,^^3^

In response, new effective pharmacological treatments, like glucagon-like peptide-1 receptor agonists (GLP1-RA), have emerged. These pharmaceuticals help regulate blood glucose levels and appetite.^4^ Clinical trials have demonstrated its efficacy, with average body weight loss of up to 15%, surpassing previous pharmacological options.^5^ Additionally, GLP1-RA have shown metabolic benefits, such as improved blood glucose levels and blood pressure^6^ as well as less sleep apnea,^7^ making it a promising therapy for individuals with obesity and related comorbidities.

Semaglutide is currently the only GLP1-RA approved for the use in weight loss in Denmark, marketed as Wegovy®. However, even in Denmark, where healthcare services are largely tax-funded and universally accessible, semaglutide for weight loss is not publicly reimbursed and must be paid out-of-pocket. This creates potential financial treatment barriers, particularly for individuals with lower income, raising concerns about unequal access to this therapy.

Though the prevalence of obesity has increased for the whole population, it is more prevalent among individuals of lower social status.^8^ However, the relationship between income and obesity is less clear. This raises important questions about how economic factors shape both the burden of obesity and access to its treatments.

This study aims to investigate the association between socioeconomic factors and use of semaglutide for weight loss in Denmark.

## Methods

### Study setting, population, and design

This nationwide register-based cross-sectional study included all Danish residents between the ages of 18 and 100 years who resided in Denmark in the entire year of 2023, thus residents who died or emigrated during 2023 were excluded. Further exclusion criteria were immigration after 1st of January 2013, history of any diagnosis of diabetes, or use of a glucose-lowering medication within the last 10 years as defined in Supplementary Table S1. We chose to exclude individuals with diabetes as they could receive semaglutide as a part of their diabetes care, which would be included in the maximum out-of-pocket costs for medication and could thus be covered by the public, potentially meaning they were less likely to receive semaglutide for weight-loss.

### Data sources and setting

Upon birth or immigration, all Danish residents are assigned a unique civil personal registration number. This number enables linkage and collection of data from the Danish nationwide registers, where information is administratively recorded on a national level for economic, social, and healthcare purposes. Both the Danish healthcare and education systems are tax-funded, providing services to all residents free of charge.

One exception is the cost of medication outside the hospital, for which people pay out-of-pocket up to a certain maximum cost for each year (approximately 600 €). However, the cost of semaglutide specifically for the indication of isolated weight loss (Wegovy®), with an estimated price of 80 € per week at maintenance dose, is not included in this maximum cost and is thus paid out-of-pocket.^9^ There was an option for the general practitioner to apply for re-imbursement by public funds through an individual application and assessment in rare cases of life-threatening illness caused by obesity.

In this study, we obtained information on individuals' age, sex, vital status, and migration from the Danish Civil Registration System. Data on hospitalizations and related diagnoses since 1973 were retrieved from the Danish National Patient Register. Procedures were identified through administrative codes using The Nordic Classification of Surgical Procedures (NCSP), see Supplementary Table S1. The Danish National Prescription Register was used to obtain information on all redeemed prescription medication in Denmark since 1995.^10^ Information on family equivalised income was obtained from the Danish Income Statistics Register.^11^ Finally, we used the Population Education Register to obtain information on the highest achieved education level.

#### Subpopulation with weight and height

Since weight and height is not routinely recorded in any Danish register, this information was not available for all individuals. However, we obtained weight and height measurements for a smaller subpopulation from The Copenhagen General Population Study.^12^^,^^13^ The Copenhagen General Population Study included individuals aged 20–100 years who were invited as a random sample from the national Danish Civil Registration System to reflect the Danish general population. We used weight and height to calculate body mass index (BMI). Obesity was defined as a BMI >30 kg/m^2^ and was subdivided further into the following groups: 30–35 kg/m^2^, 35–40 kg/m^2^, and >40 kg/m^2^.

### Covariates

#### Income

Statistics Denmark provides an income measure called equivalised income, which ensures comparability between different households by accounting for family size. Equivalised income is based on the Organization for Economic Cooperation and Development (OECD) modified scale, which weights and redistributes a household's total disposable income equally among all family members, giving them the same equivalised income.^11^ In this study, we obtained the equivalised income for each individual for 2023 and divided it into four quartiles, ranging from the lowest to the highest equivalised income. These data were registered at an individual level but depend on data availability for the complete family. In sensitivity analyses we looked at the rate of prescription for individuals without income data both as a group and as if they were of the highest income quartile.

#### Education level

Education level was defined as highest achieved education in 2023 and the individuals were divided into the four groups based on the International Standard Classification of Education (ISCED). Group one includes primary education, and lower secondary education ISCED level 0–2. Group two represents general upper secondary education and vocational upper secondary education ISCED level 3. Group three includes short-cycle tertiary, medium-length tertiary, and bachelor's-level education or equivalent, ISCED level 5–6. Group four represents second-cycle, master's-level or equivalent and PhD-level, ISCED level 7–8. We did not use ISCED level 4, as no education in Denmark is classified to this level.

#### Immigration status

The individual immigration status was classified as either ethnic Danes or immigrants/descendants following the definitions by Statistics Denmark. An ethnic Dane is defined as a person with at least one parent originating from Denmark and has Danish citizenship. An immigrant is defined as a person born outside of Denmark with parents who were also born outside of Denmark. A descendant is defined as a person born in Denmark whose parents were both born outside of Denmark.

#### Comorbidities

Comorbidities were identified based on all primary and secondary diagnoses coded according to the International Classification of Diseases, Tenth Revisions (ICD-10). Comorbidities included ischemic heart disease, cancer, stroke, and chronic obstructive pulmonary disease. For further details, see Supplementary Table S1.

### Outcome

The primary outcome was redemption of at least one prescription for semaglutide for weight loss, in the following solely referred to as “prescription”. Prescription redemption was analyzed by counting the number of individuals who redeemed at least one prescription stratified by quarter of 2023. Additionally, we assessed the total number of individuals who redeemed any prescription during 2023. Semaglutide prescriptions were identified by both ATC code and item number regardless of concentration. The ATC code and item numbers used are shown in Supplementary Table S1. As a subanalysis, we investigated the association between income and bariatric surgery during 2023.

### Statistics

Characteristics of the study population were reported stratified for any redeemed prescription for semaglutide, and baseline characteristics were reported in counts (%) or median (interquartile range [IQR]). Analyses were performed based on the quarters of the year 2023 and stratified by age, sex, educational level, immigration status, comorbidities, and income. Results were reported as a percentage of the corresponding population. We used logistic regression to assess the association between redemption of semaglutide prescriptions in 2023 and age, sex, household income, educational level, immigration status, and comorbidities and reported the odds ratio (OR) with 95% confidence intervals (95% CI). All data management and statistical analysis were performed using R statistical software version 4·4·1.

### Ethics approval

In Denmark, register-based studies conducted solely for statistical and scientific research do not require approval by an ethics committee or informed consent.^14^ However, the study was conducted in compliance with both the Danish Data Protection Act and the General Data Protection Regulation (GDPR) and was approved by the data-responsible institute Capital Region of Denmark. The Copenhagen General Population Study was approved by institutional review boards and Danish ethical committees, and participants provided written informed consent.

### Role of funding source

This study received no funding.

## Results

There were 4,922,272 individuals between the age of 18 and 100 years in Denmark in 2023, and after excluding individuals without income data (204,303, 4·2%) and with diabetes (186,823, 3·8%), a total of 4,531,146 individuals were included. Baseline characteristics stratified by prescription of semaglutide for weight loss in 2023 is shown in Table 1. Individuals with at least one prescription of semaglutide were more likely to be women, less likely to be an immigrant or a descendant, more likely to be in the upper two quartiles of income, have a history of ischemic heart disease and chronic obstructive pulmonary disease but less likely to have a history of cancer.

**Table 1:** Baseline characteristics of the population stratified by any redeemed prescription of semaglutide for weight loss in 2023.

	No semaglutide	Semaglutide	p
n	4,423,919	107,227	
Women, n (%)	2,225,784 (50·3)	75,179 (70·1)	<0·001
Age, n (%)			<0·001
18–29 years	907,059 (20·5)	9222 (8·6)	
30–39 years	704,809 (15·9)	17,529 (16·3)	
40–49 years	681,038 (15·4)	27,859 (26·0)	
50–59 years	748,153 (16·9)	33,507 (31·2)	
60–69 years	621,154 (14·0)	14,350 (13·4)	
>70 years	761,706 (17·2)	4760 (4·4)	
Immigrants or descendants, n (%)	698,633 (15·8)	11,889 (11·1)	<0·001
Educational level, n (%)			<0·001
Level 1	887,722 (20·3)	17,533 (16·5)	
Level 2	1,824,955 (41·8)	47,877 (45·0)	
Level 3	1,086,489 (24·9)	30,371 (28·6)	
Level 4	567,157 (13·0)	10,553 (9·9)	
Income quartile, n (%)			<0·001
Lowest (<195,956 DKK)	1,099,568 (24·9)	13,925 (13·0)	
2. Lowest (195,956–273,678 DKK)	1,087,404 (24·6)	20,861 (19·5)	
2. Highest (273,678–372,843 DKK)	1,116,278 (25·2)	31,143 (29·0)	
Highest (>372,843 DKK)	1,120,669 (25·3)	41,298 (38·5)	
IHD, n (%)	79,621 (1·8)	1931 (1·8)	0·989
Cancer, n (%)	155,819 (3·5)	3133 (2·9)	<0·001
Stroke, n (%)	107,469 (2·4)	2058 (1·9)	<0·001
COPD, n (%)	51,665 (1·2)	1165 (1·1)	0·015

IHD—Ischemic heart disease, COPD—Chronic obstructive pulmonary disease, DKK—Danish Kroner (1 DKK = 0·13 €).

**Fig. 1:**
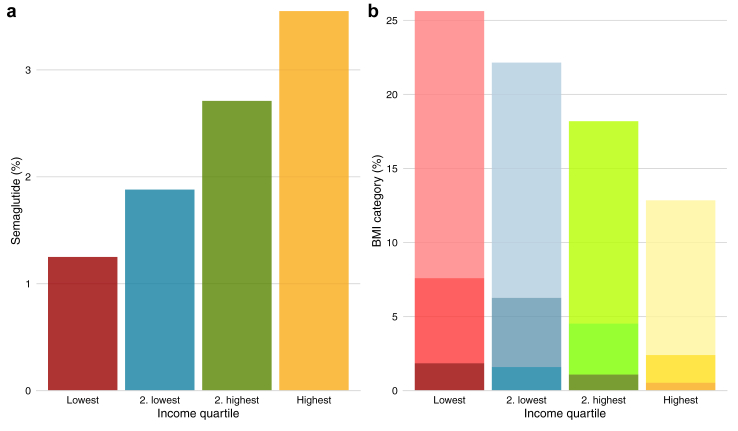
Panel a) Percentage of individuals with a redeemed prescription of semaglutide for weight loss stratified by income quartile. Panel b) Proportion of individuals in categories classified as overweight included in The Copenhagen General Population Study (n = 36,391) stratified by income quartile. Colors indicate income and opacity indicates body mass index category (BMI). Darkest colors—BMI >40, middle opacity—BMI 35–40, lightest colors—BMI 30–35.

The proportion of individuals with at least one prescription of semaglutide stratified by income quartile is shown in Fig. 1 panel a. We saw a progressive increase in the proportion of individuals with a prescription with increasing income from 1·3% in the lowest income quartile to 3·6% in the highest income quartile. Fig. 1 panel b shows the distribution of BMI categories by income in the subpopulation (n = 36,391) included in The Copenhagen General Population Study. Here we saw the proportion with obesity decline from 26% in the lowest income quartile to 13% in the highest income quartile. This was consistent across sex, as shown in Supplementary Figure S1. The sensitivity analyses of individuals without registry data on income, showed that they had a low rate of prescription, for any prescription in 2023 it was 307 (0·2%). However, when combined with individuals in the highest income quartile it did not change the association between income and prescription, see Supplementary Figure S2.

**Fig. 2:**
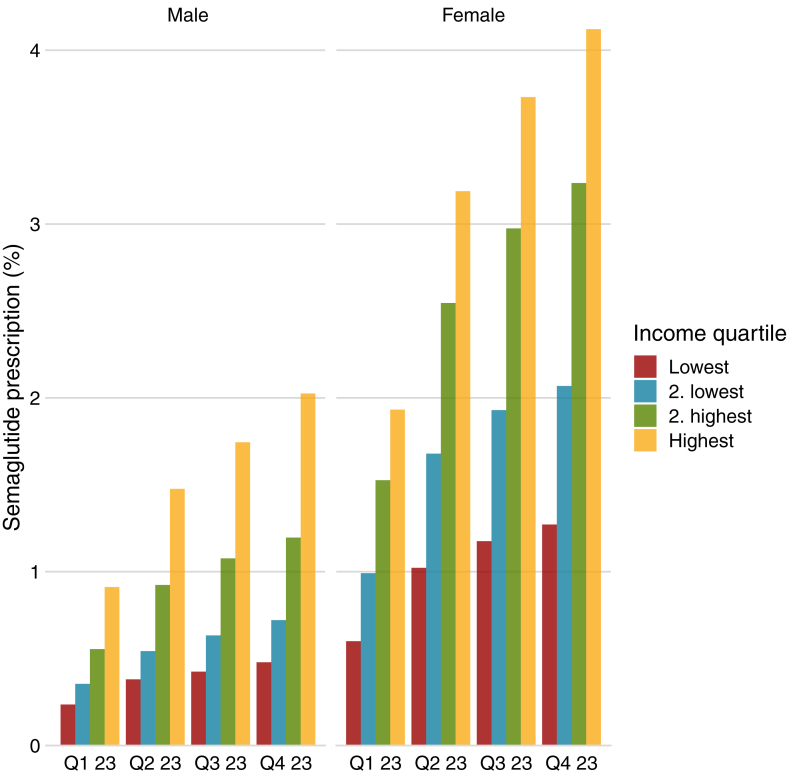
Percentage of individuals with a redeemed prescription of semaglutide for weight loss stratified by quarter of the year 2023, sex, and income quartile. Q—Quarter of 2023.

Overall, there was more than a doubling of the frequency of individuals in 2023 with a prescription over the year from 40,605 (0·9% of all included) in the first quarter of 2023 to 86,431 (1·9% of all included) in the fourth quarter. The frequency of prescriptions for each quarter of 2023 stratified by sex and income is shown in Fig. 2. This showed an increase in prescription for all groups. Women in the highest quartile of income had the highest increase from 10,818 (1·9% of women) to 23,069 (4·1% of women) prescriptions between the first and fourth quarter of 2023.

**Fig. 3:**
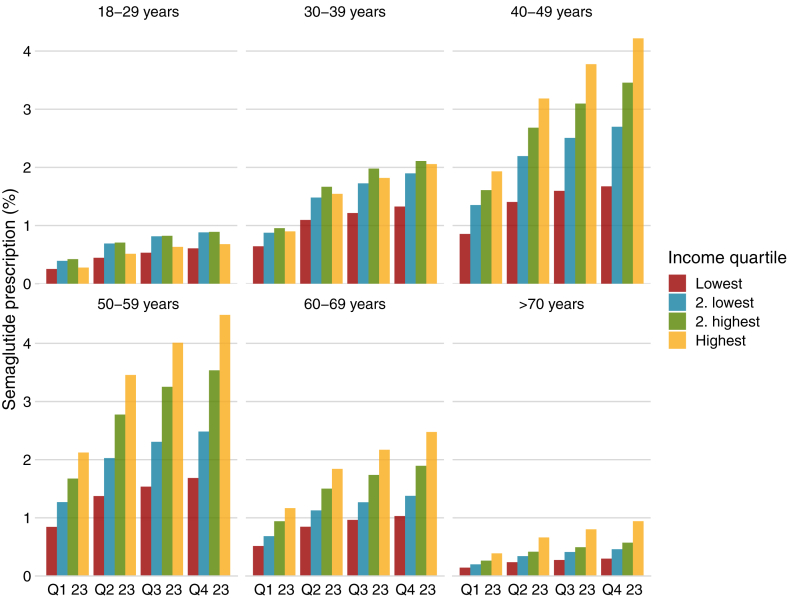
Percentage of individuals with a redeemed prescription of Semaglutide stratified by quarter of the year 2023, age, and income quartile. Q—Quarter of 2023.

**Fig. 4:**
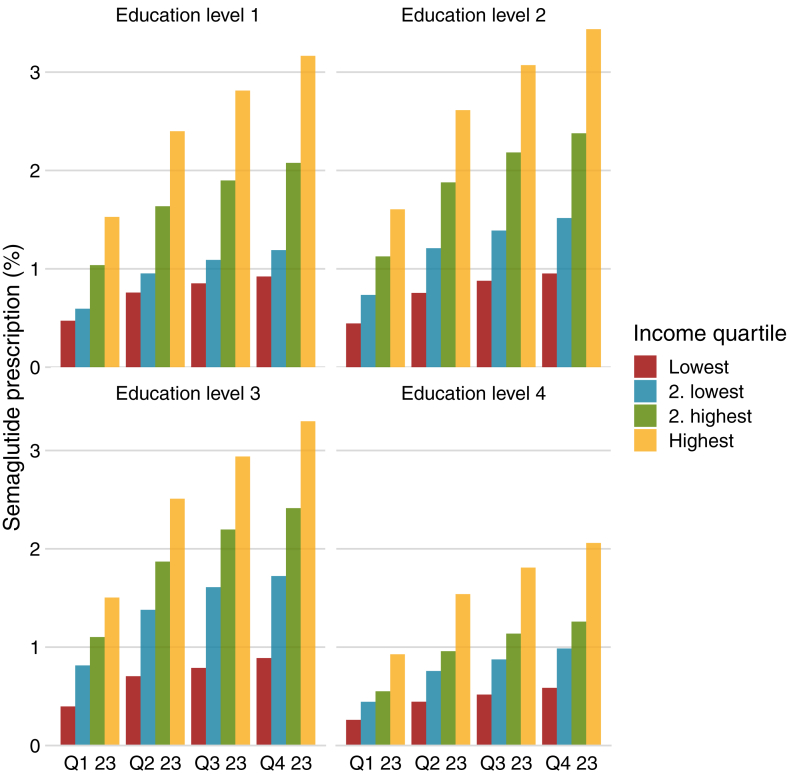
Percentage of individuals with a redeemed prescription of semaglutide stratified by quarter of the year 2023, educational level and income quartile. Q—Quarter.

**Fig. 5:**
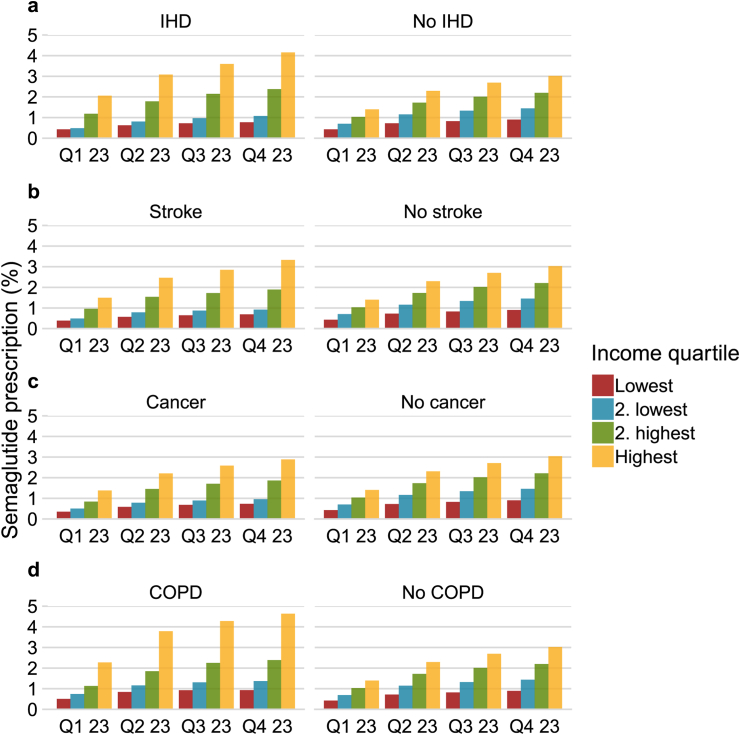
Percentage of individuals with a redeemed prescription of semaglutide stratified by quarter of the year 2023, income quartile and comorbidities. Panel a is stratified by ischemic heart disease (IHD), panel b is stratified by stroke, and panel c by cancer, and panel d by chronic obstructive pulmonary disease. Q—Quarter, IHD—Ischemic heart disease, COPD—Chronic obstructive pulmonary disease.

Fig. 3 shows the proportion of prescriptions stratified by age and income. The age groups with most prescriptions were middle aged individuals 40–49 years and 50–59 years whereas prescriptions were rare among individuals >80 years. There was a strong association between income and prescriptions for all but the youngest age group. Fig. 4 shows the prescription frequency stratified by educational level and income for individuals with data on education (n = 4,472,657; 98·7% of all). The association between income and prescription was consistent across educational groups. However, while there were a similar proportion of prescriptions for educational levels 1, 2, and 3, individuals in the highest educational group were less likely to use semaglutide in all income groups. Fig. 5 show the prescription frequency in relation to comorbidities, these show that ischemic heart disease, chronic obstructive pulmonary disease, and stroke were related to a higher frequency of prescription compared to those without the disease while cancer had a reverse relation to prescription of semaglutide.

The results of the logistic regression model for any prescription of semaglutide in 2023 is shown in Supplementary Figure S2. We found that there was a strong association between income and semaglutide use with individuals in the highest income quartile having an OR of 2·10 (95% CI 2·05–2·14) compared to individuals in the lowest income quartile when adjusting for age, sex, immigration status, educational level, and comorbidities.

In our subanalysis of bariatric surgery, we found that the procedures were rare compared to prescription of semaglutide for weight-loss, with a total of 1834 procedures (0·04% of all individuals) in 2023, and was most common for individuals in the 2. Highest income quartile and 2. Lowest income quartile, see Supplementary Figure S3.

## Discussion

This study showed a strong positive association between income and use of semaglutide, as individuals in the highest income quartile consistently had the highest usage frequencies across all quarters of 2023. This pattern persisted even when stratified by sex, age, and education. Despite this, when investigating the association between income and obesity we saw the inverse relationship, with lower rates of obesity among individuals with a higher household income. This indicates that financial barriers may limit access to obesity treatment for lower-income groups in Denmark. We found that the use of semaglutide was highest in women and individuals in the ages 40–60 years. Additionally, having a history of stroke, ischemic heart disease or chronic obstructive pulmonary disease was associated with increased use of semaglutide, while cancer was not. However, adjusting for comorbidities did not change the strong association between income and prescriptions of semaglutide for weight-loss. The association between comorbidities and use of weight-loss drugs should be studied further in the future. Interestingly, the association between educational level and semaglutide use was inconsistent, with the highest educated individuals being less likely to use semaglutide. We found an association between income and bariatric surgery with a lower rate in the lowest and highest income quartile. Bariatric surgery is a free procedure for the patient, while semaglutide is paid by the individual. Whether the introduction of semaglutide for weight-loss will change the population having bariatric surgery, e.g., a larger proportion being in the lower income group should be investigated further as more data is collected.

Despite the Danish universal healthcare system, the out-of-pocket cost of semaglutide, which is currently not covered by public medication reimbursement, disproportionately affects those with fewer financial resources. The cost of semaglutide for weight loss is high compared to other drugs, even in comparison to semaglutide used for diabetes management.^9^ This may create a paradox where those who need weight loss treatments the most have limited access.

The marked effect of income on semaglutide in a high-income country such as Denmark implies similar, if not worse, issues in lower-income countries with healthcare systems with a higher proportion of medical expenditure being out-of-pocket. Future studies should clarify the effect of income inequality on access to effective weight-loss treatment in these settings.

Notably, our data indicates that women have a higher usage of semaglutide compared to males. This trend may reflect greater health-seeking behavior among women, particularly concerning weight management.^15^^,^^16^ Another possible factor could be the increased weight-based stigmatization among women.^17^ This could indicate that cosmetic factors are important drivers of semaglutide use, and a survey of members of The Aesthetic Society in July 2023 showed that nearly a third reported using semaglutide as a part of their treatment.^18^ Potential overtreatment due to cosmetic considerations should be explored in the future as it could lead to harm, with the most notable side effects of GLP1-RA being gastro-intestinal, and Non-Arteritic Anterior Ischemic Optic Neuropathy being a rare but serious adverse effect.^19^^,^^20^

Likewise, age stratified analysis revealed that semaglutide usage peaked for individuals aged 40–49 years, which could reflect a higher disposable income in these age groups. Studies have demonstrated that obese, higher income individuals are more likely to receive weight loss advice from health care providers.^21^

Obesity is a risk factor for development of diabetes, cardiovascular diseases, cancer and a variety of other chronic diseases as well as premature death.^1^^,^^3^^,^^22^^,^^23^ Mitigating these risks through effective therapies have been difficult, but newer drugs such as semaglutide have proven effective for weight loss.^5^ Given the possible reductions in disease risk attributable to weight loss, increasing availability of these new drugs could lead to overall improvements in health. However, the combination of high sales prices and lack of reimbursement by the national health system for these drugs seems to limit the access to this treatment.

Previous studies have shown associations between socioeconomic status and obesity in developed countries with obesity being more prevalent in populations with lower socioeconomic status, especially for women.^24^^,^^25^ Furthermore, people with obesity are already at a financial disadvantage since medical expenses in this population are higher compared to people not living with obesity.^26^ This provides the substrate for a vicious cycle, where people living with obesity at lower income levels are driven further into poverty and social stigmatization, reducing their resources and chances of weight loss, which in return yields higher disease burdens and increased medical expenses. Breaking this cycle could be an incentive for government initiatives to target these populations for reimbursement on medical expenses for weight loss drugs, since these people would be given a better opportunity at a healthier and more prosperous life. Which would contribute to more equality in health across society and even reduce state health care expenses, as reports also claim that obesity places an excess financial burden of up to 2·8% on health care systems.^26^

Previous literature highlights the complex and multifactorial relationship between income and access to weight-loss treatment.^27^ Socioeconomic disparities in care are further exacerbated by weight-related stigma, which contributes to the underutilization of effective therapies. A systematic review of weight-loss and weight-loss maintenance trials found that the majority of studies failed to account for structural inequalities,^28^ most likely due to limited sample sizes and insufficient attention to social determinants of health. Echoing our findings on semaglutide use, the review also reported that women and older individuals were more frequently represented in weight management trials. Notably, however, when men were explicitly invited to participate, their enrollment rates matched those of women, suggesting that targeted engagement strategies may help reduce sex-based disparities in the care of individuals living with obesity. Our study is based on the Danish National Prescription Registry,^10^ a well-validated source of administrative data that captures all prescriptions across Denmark. A key strength of this registry is that it records redeemed, not merely issued, prescriptions. This distinction is especially important in the context of semaglutide, which is a costly medication. By focusing on redemption, we are able to minimize the impact of primary non-adherence, a phenomenon likely to be more pronounced among individuals facing financial barriers.^29^ Consequently, the use of redeemed prescription data provides a more accurate estimate of actual medication uptake and strengthens our ability to assess socioeconomic disparities in real-world use. Furthermore, the registry includes prescriptions from both public and private prescribers, ensuring comprehensive national coverage.

We used equivalized income to estimate individual disposable income, as it accounts for the total household income adjusted for household composition. This approach provides a more accurate reflection of the individual's economic resources by incorporating income from all household members and applying standardized weights based on household size. Importantly, this method mitigates the risk of misclassifying individuals, such as low- or non-earning partners in high-income households, as low-income when only personal income is considered.

While semaglutide in the formulation investigated in this study is approved in Denmark for weight loss treatment, prior data suggest that another formulation of semaglutide, approved only for type 2 diabetes, have also been used off-label for weight loss.^30^ However, these data were primarily from before the release of semaglutide for weight loss in Denmark. During the study period formulations for type 2 diabetes were similarly priced, only available at lower doses, and like the formulation for weight-loss they were not reimbursed for the treatment of obesity. We excluded individuals with diabetes as they could receive semaglutide as a part of their diabetes care. We chose a restrictive definition of diabetes to minimize the risk of bias as individuals at risk of being diagnosed with diabetes but never treated could potentially receive semaglutide for weight-loss as a part of their care. This may have led us to underestimate the prevalence of diabetes.

Similarly, we did not stratify by dosage of semaglutide in this study.

Since 1st of January 2024 the largest private health insurance company (Sygeforsikring Denmark) removed the reimbursement of semaglutide from their health insurance plans as the cost was too high. How this may have influenced the use among the lower income groups after our inclusion period is unclear. We did not have access to height and weight data for the entire Danish population, which precluded direct assessment of the relationship between BMI and semaglutide prescriptions at the national level. Instead, we used data from the Copenhagen General Population Study (CGPS), collected between 2014 and 2019. CGPS is a well-characterized and population-based cohort designed to reflect the Danish adult population.^12^^,^^13^ However, demographic and epidemiological shifts over the intervening years may limit full comparability to the full Danish population in 2023. Moreover, we defined obesity as BMI >30 kg/m^2^ in line with standard definitions. However, semaglutide for weight-loss can be prescribed to individuals with a BMI between 27 and 30 kg/m^2^ with relevant comorbidities. This discrepancy should be considered when interpreting the results. A major strength of this register-based study was the use of high-quality data collected for administrative purposes, which enhances information validity and minimizes loss to follow-up. Additionally, the inclusion of all Danish citizens reduces the risk of selection bias, ensuring representation across all ages, sexes, ethnicities, and socioeconomic groups.

### Conclusion

We found a strong association between high income level and use of semaglutide for weight loss. This difference may potentially exacerbate existing health inequalities.

## Contributors

RBH, MPA, MKA contributed equally to the conceptualization. Data curation was performed by RBH and MKA, with support from KKS. The formal analysis was performed by RBH and MKA, with support from KKS. The methodology was chosen by RBH, MPA, and MKA, with support from CN, MH, and PDWY. CTP and MPA contributed resources with support from BGN, SA, and CSD. Supervision was provided by SNFB, HCC, CG, SA, BGN, KK, HB, KKI, and CTP. The original draft was written by RBH, MPA, and MKA, with support from CN, MH, and PDWY. RBH, MPA, and MKA had access to the raw data. RBH, MPA, and MKA verified the data. All authors critically reviewed the study, revised the manuscript, gave final approval and agree to be accountable for all aspects of the work ensuring integrity and accuracy. All authors share the final responsibility for the decision to submit for publication.

## Data sharing statement

Data were accessed on protected servers at Statistics Denmark. Only Danish research environments are granted authorization from Statistics Denmark. However, foreign researchers can get access through an affiliation to an authorized research environment.

## Declaration of interests

Børge Nordestgaard reports receiving consulting fees from Novo Nordisk and Lilly and having participated on advisory board for Novo Nordisk and Lilly. Rasmus Bo Hasselbalch reports support from the Novo Nordisk Foundation for work outside this study. Cristian Torp-Pedersen reports receiving grants from Novo Nordisk and Bayer for work outside this study. Kristian Kragholm reports receiving grants from Novo Nordisk Foundation and The Danish Cancer Society outside this work. Kristian Kragholm reports receiving speaker honoraria from Novo Nordisk. All other authors report no relevant disclosures.

## Appendix A Supplementary data

Supplementary Materials

STROBE Checklist
