# Supplementary material for: Association between socioeconomic factors and semaglutide use for weight loss: a population-based cross-sectional study in Denmark
Source: Lancet Reg Health Eur. 2025 Jul 28;56:101398. doi: 10.1016/j.lanepe.2025.101398 (PMC12859550; doi:10.1016/j.lanepe.2025.101398)

Supplementary materials

Association between socioeconomic factors and semaglutide use for weight loss: a population-based cross-sectional study in Denmark

Contents

Supplementary methods and references.. 3

Supplementary Table S1 – Conditions and diagnosis codes 4

Supplementary figure 1 – Panel A) Percentage of individuals with a redeemed prescription of semaglutide for weight loss stratified by sex and income quartile. Panel B) Proportion of individuals in categories classified as overweight included in The Copenhagen General Population Study (n=36,391) stratified by sex and income quartile. Colors indicate income and opacity indicates body mass index category (BMI). Darkest colors – BMI >40, middle opacity – BMI 35-40, lightest colors – BMI 30-35. 5

Supplementary figure 2 – Panel a) Percentage of individuals with a redeemed prescription of semaglutide for weight loss stratified by income quartile including individuals without income data (NA). Panel b) Percentage of individuals with a redeemed prescription of semaglutide for weight loss stratified by income quartile when including individuals without income data in the highest income quartile. 6

Supplementary figure 3 – Percentage of individuals who underwent bariatric surgery in 2023 stratified by income quartile... 7

Supplementary figure 4 – Forest plot of logistic regression for any semaglutide prescription in 2023. IHD – Ischemic heart disease, COPD – Chronic obstructive pulmonary disease... 8

Supplementary methods and references

We obtained information on individuals' age, sex, vital status, and migration from the Danish Civil Registration System(1).

Data on hospitalizations and related diagnoses since 1973 were retrieved from the Danish National Patient Register(2).

Education level was obtained from we the Population Education Register to obtain information on the highest achieved education level(3) and the educational levels were defined based on the International Standard Classification of Education (ISCED)(3).

An immigrant is defined as a person born outside of Denmark with parents who were also born outside of Denmark. A descendant was defined as a person born in Denmark whose parents were both born outside of Denmark(4).

Comorbidities were identified based on all primary and secondary diagnoses coded according to the International Classification of Diseases, Tenth Revisions (ICD-10)(5).

All data management and statistical analysis were performed using R statistical software version 4.4.1(6).

References

1. Schmidt M, Pedersen L, Sørensen HT. The Danish Civil Registration System as a tool in epidemiology. Eur J Epidemiol. 2014;29(8):541–9.

2. Schmidt M, Schmidt SAJ, Sandegaard JL, Ehrenstein V, Pedersen L, Sørensen HT. The Danish National Patient Registry: a review of content, data quality, and research potential. Clin Epidemiol [Internet]. 2015 Nov 17 [cited 2022 Mar 24];7:449–90. Available from: https://pubmed.ncbi.nlm.nih.gov/26604824/

3. Jensen VM, Rasmussen AW. Danish education registers. Scand J Public Health. 2011;39(7):91–4.

4. Statistics Denmark. Documentation of statistics for Immigrants and Descendants 2017 Month 01 [Internet]. 2017. Available from: https://www.dst.dk/Site/Dst/SingleFiles/GetArchiveFile.aspx?fi=131437185074&fo=0&ext=kvaldel

5. Schmidt M, Schmidt SAJ, Adelborg K, Sundbøll J, Laugesen K, Ehrenstein V, et al. The Danish health care system and epidemiological research: from health care contacts to database records. Clin Epidemiol [Internet]. 2019;11:563–91. Available from: http://www.ncbi.nlm.nih.gov/pubmed/31372058

6. Team RC. R: A Language and Enviroment for Statistical Computing [Internet]. 2016. Available from: https://www.r-project.org

Supplementary Table S1 – Conditions and diagnosis codes

Condition	Subgroups included	ATC codes	ICD-10 codes
Diabetes		A10 except A10BJ and A10BX	E10-14
Ischemic Heart Disease	Myocardial infarction, Chronic myocardial ischemia		I21-25
Stroke			I60-66, I678-679, I69, G45
Chronic Obstructive Pulmonary Disease			J41-44
Cancer	Cancer, metastases, lymphoma, leukaemia, melanoma		C00-43, C45-97

Medication	ATC code	Item numbers
Semaglutide for weight loss (Wegovy®)	A10BJ06	468849, 409364, 386270, 178249, 153271, 437102, 409687, 191797, 187574, 066923, 405773, 394103, 191243, 131824, 128420, 058102, 560545, 538908, 534716, 481461, 396934, 395234, 448583, 431609, 418253, 178307, 164303
Procedures	Bariatric surgery	KJDF1, KJDF2, KJDF4

Supplementary figure 1 – Panel A) Percentage of individuals with a redeemed prescription of semaglutide for weight loss stratified by sex and income quartile. Panel B) Proportion of individuals in categories classified as overweight included in The Copenhagen General Population Study (n=36,391) stratified by sex and income quartile. Colors indicate income and opacity indicates body mass index category (BMI). Darkest colors – BMI >40, middle opacity – BMI 35-40, lightest colors – BMI 30-35.

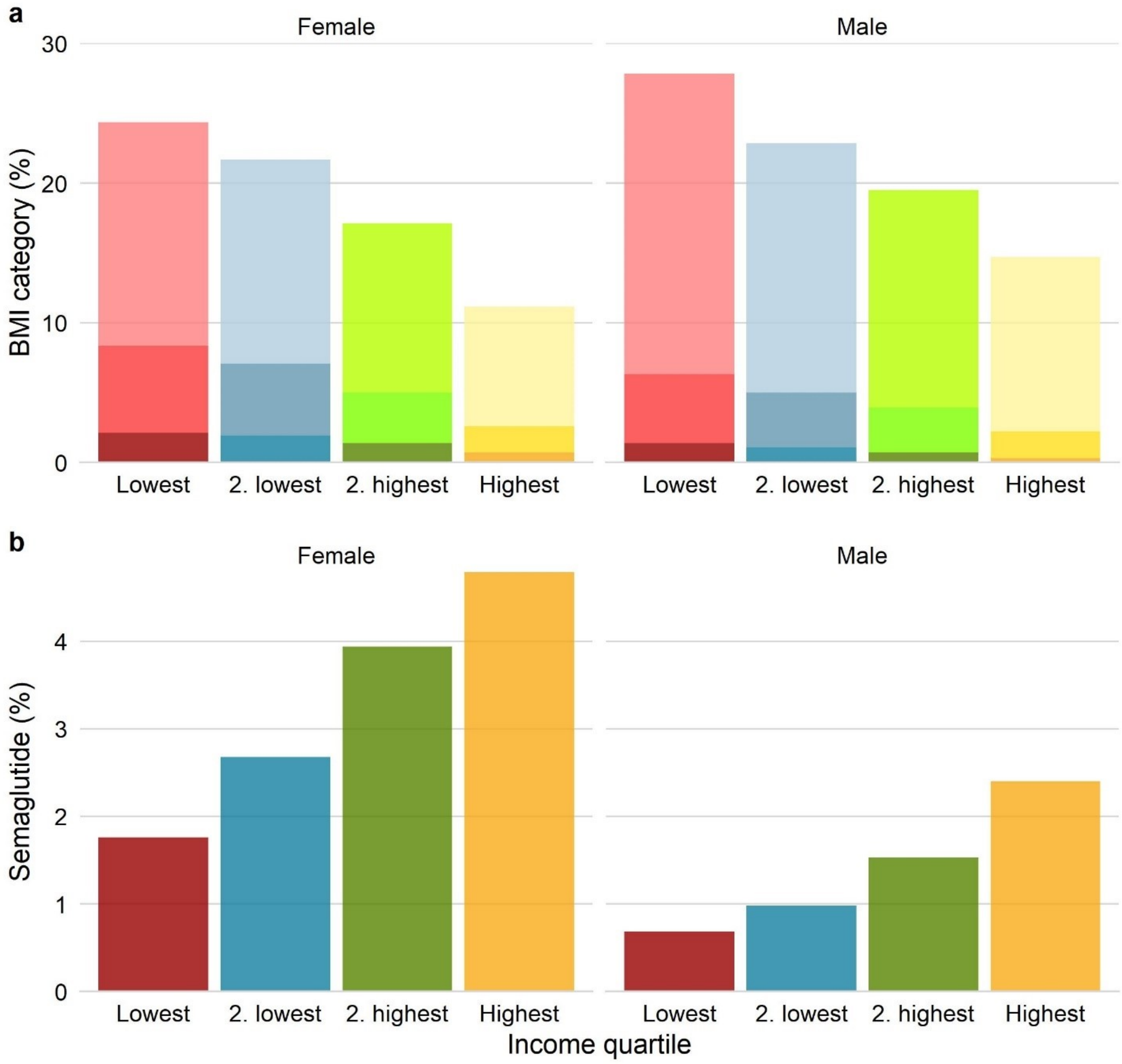

Supplementary figure 2 – Panel a) Percentage of individuals with a redeemed prescription of semaglutide for weight loss stratified by income quartile including individuals without income data (NA). Panel b) Percentage of individuals with a redeemed prescription of semaglutide for weight loss stratified by income quartile when including individuals without income data in the highest income quartile.

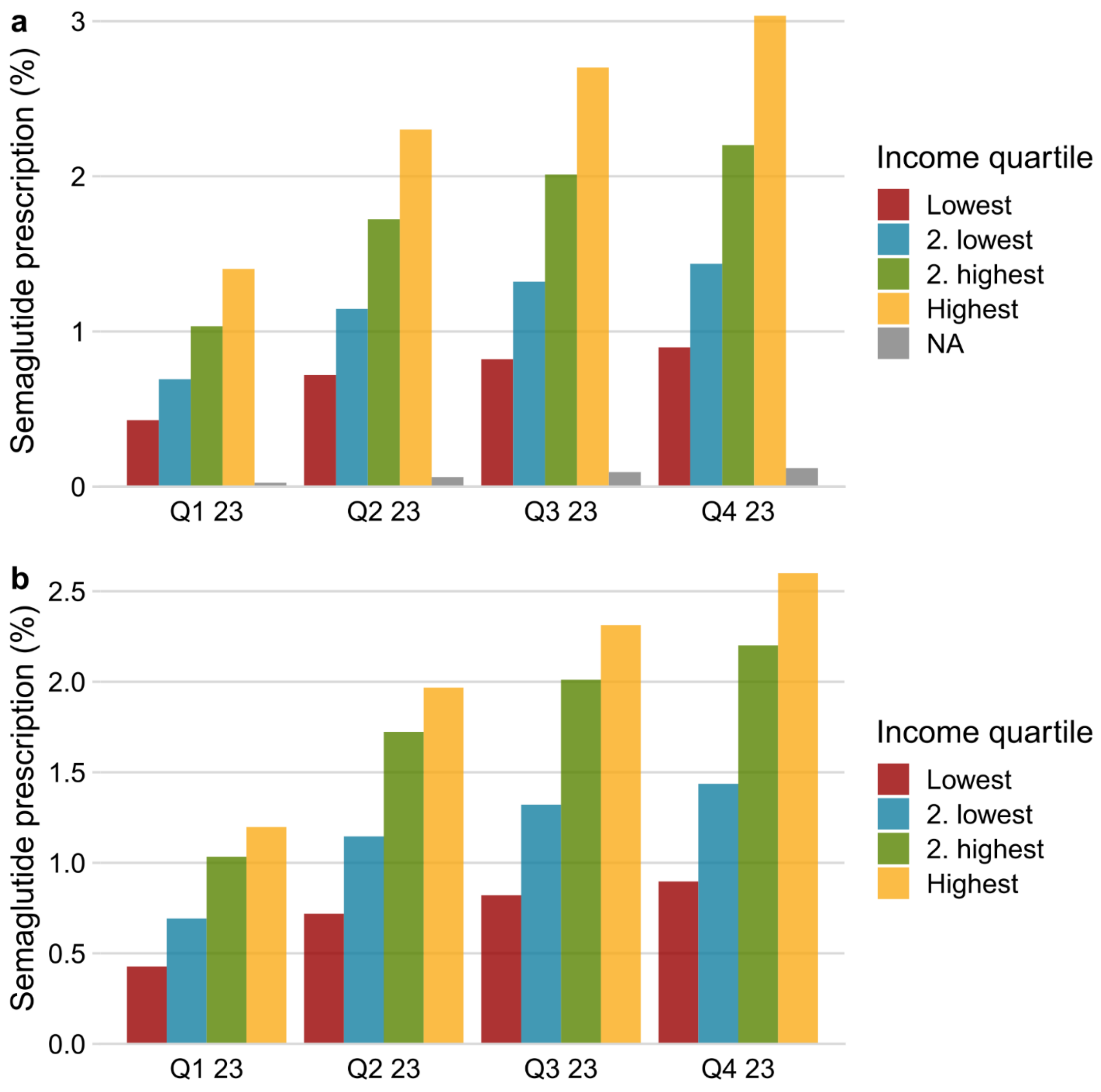

Supplementary figure 3 – Percentage of individuals who underwent bariatric surgery in 2023 stratified by income quartile.

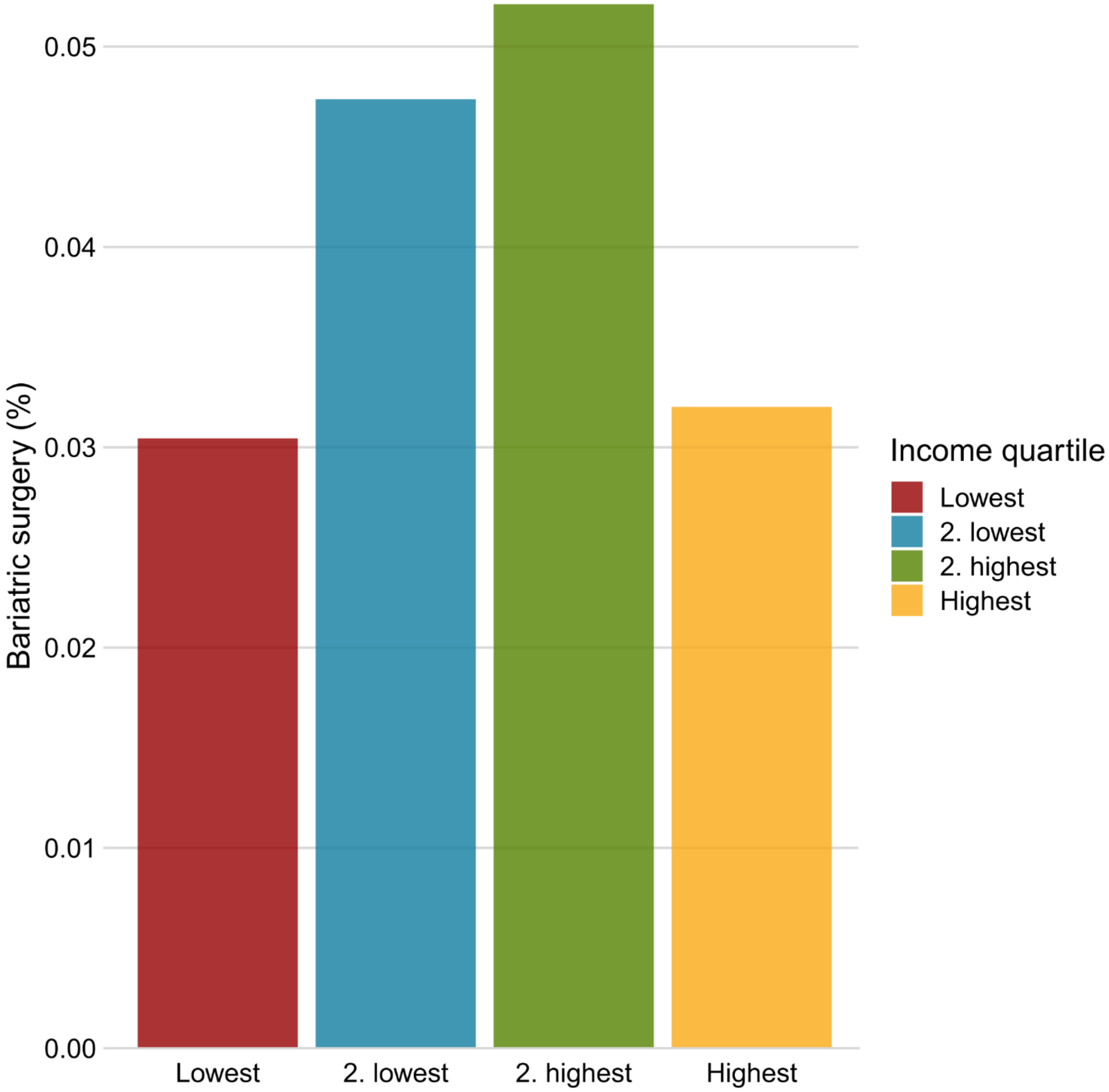

Supplementary figure 4 – Forest plot of logistic regression for any semaglutide prescription in 2023. IHD – Ischemic heart disease, COPD – Chronic obstructive pulmonary disease.

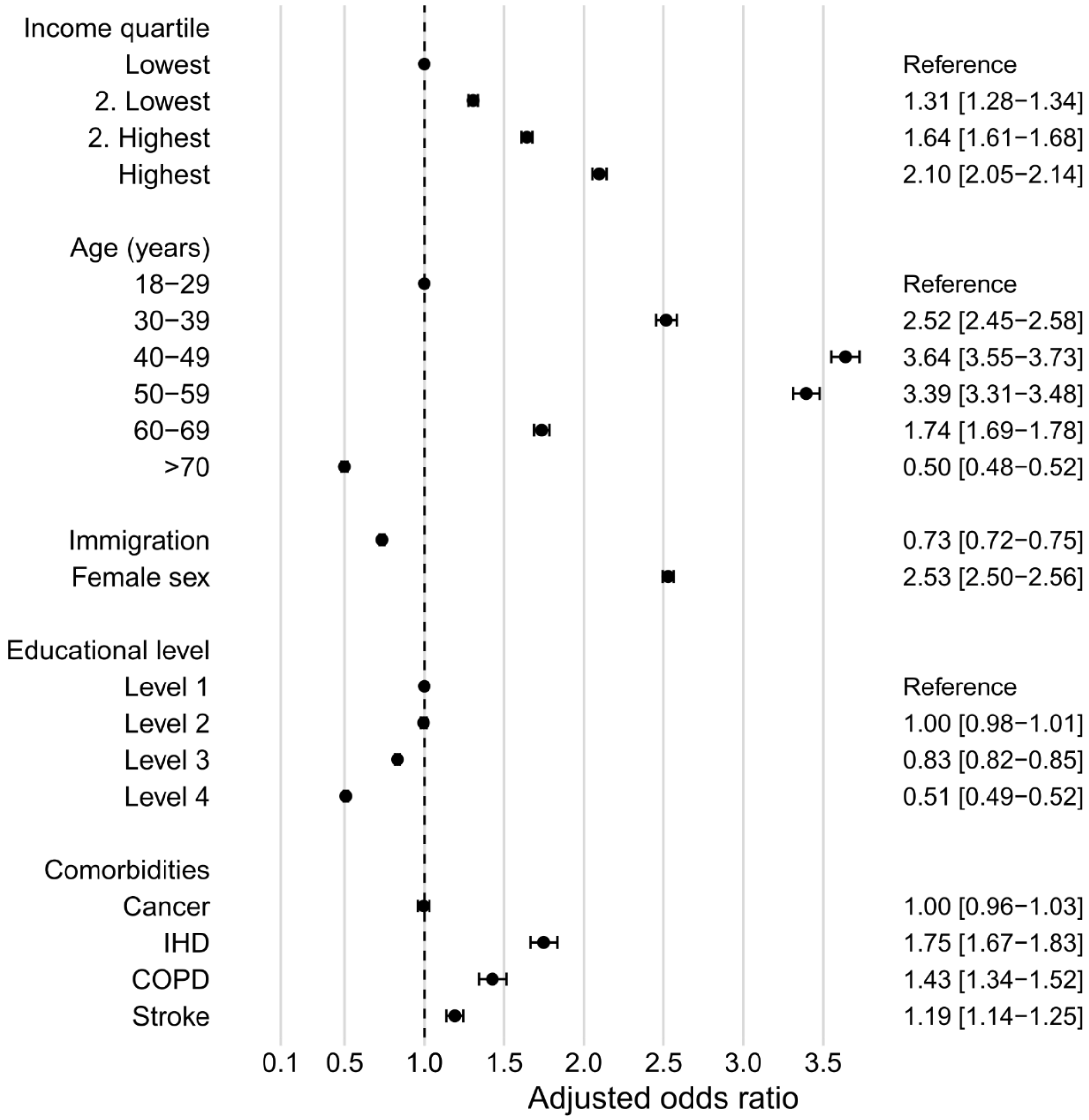

Supplement: Supplementary Materials [file mmc1.pdf]
